# Possibilities of Improving the Clinical Value of Immune Checkpoint Inhibitor Therapies in Cancer Care by Optimizing Patient Selection

**DOI:** 10.3390/ijms21020556

**Published:** 2020-01-15

**Authors:** Sanna Iivanainen, Jussi P. Koivunen

**Affiliations:** Department of Oncology and Radiotherapy, Oulu University Hospital and MRC Oulu, 90220 Oulu, Finland; sanna.iivanainen@ppshp.fi

**Keywords:** cancer immunotherapy, immune checkpoint inhibitors, predictive, biomarker, cost-effectiveness, immune-related adverse events, PD-L1

## Abstract

Immune checkpoint inhibitor (ICI) therapies have become the most important medical therapies in many malignancies, such as melanoma, non-small-cell lung cancer, and urogenital cancers. However, due to generally low response rates of PD-(L)1 monotherapy, both PD-(L)1 combination therapies and novel therapeutics are under large-scale clinical evaluation. Thus far, clinical trials have rather suboptimally defined the patient population most likely to benefit from ICI therapy, and there is an unmet need for negative predictive markers aiming to reduce the number of non-responding patients in clinical practice. Furthermore, there is a strong need for basic tumor immunology research and innovative clinical trials to fully unleash the potential of ICI combinations for the benefit of patients.

## 1. Introduction

The basis for cancer immunotherapy was obtained in the 1940s when the observation that mainly CD8+ cytotoxic T cells, a subtype of lymphocytes, were responsible for mediating the rejection and led to the identification of the mechanistic basis of antigen-recognition by human tumor-reactive T cells. In the past 10 years, there has been a huge development in cancer immunotherapies with the introduction of immune checkpoint inhibitor therapies towards programmed cell death protein 1 (PD-1), programmed death ligand-1 (PD-L1), and cytotoxic T-lymphocyte-associated antigen 4 (CTLA-4) [1]. Immune checkpoint inhibitor (ICI) therapies have become the most important medical therapies in many malignancies such as melanoma, non-small-cell lung cancer, and urogenital cancers [2,3,4,5,6,7,8,9,10,11]. The mechanism of action of ICIs is in the inhibition of T-cell blocking, which can result in increased T-cell mediated cancer cell death [1].

Anti-PD-(L)1 antibodies have been approved for numerous metastatic cancers and recently in adjuvant settings after operation of stage III melanoma or chemoirradation of stage III non-small-cell lung cancer (NSCLC) [2,5,6,7,8,9,10,11,12,13,14,15,16]. Despite the continuously evolving indications of ICIs, clinical knowledge about their efficacy and tolerability in real-world settings is inadequate [17,18,19]. Typically, responses are seen in 20–40% of the metastatic cancer patients treated with PD-(L)1 monotherapy, yet most fail to respond [20,21,22,23]. Reproducible predictive biomarkers for anti-PD-(L)1 response are lacking. Due to generally low response rates of PD-(L)1 monotherapy, both PD-(L)1 combination therapies and novel therapeutics are under large-scale clinical evaluation. Novel immunotherapeutic drugs combined to PD-(L)1 antibodies including agents targeting T-cell receptors (e.g., LAG-3 [24], OX40 antibodies; NCT03971409), macrophages (e.g., CSF1R, CD47 antibodies [25]), and cytokines (e.g., IL-β1; NCT03631199) are under wide investigation. In this review, we focus on the current clinical concepts and future perspectives of anti-PD-(L)1 therapies in cancer care.

## 2. Cancer Immunotherapy

### 2.1. The Rationale of T Cell Focused Therapies

T cells are able to home into the antigen-expressing tumor bed, proliferate in response to tumor antigens, eradicate tumor cells by direct cytotoxicity, and generate immunological memory that allows a response to reoccurring tumors [26]. These features make T-cell focused therapies mechanistically preferable compared to other forms of cancer immunotherapy. In addition to cytotoxic CD8+ T-cells, the tumor microenvironment (TME) also contains other immunological cells that can support or inhibit cytotoxic T-cell functions. Specific CD4+ T helper (Th) cell phenotypes are essential for the efficient effector function of CD8+ T cells. Th1 CD4+ T cells secrete type I cytokines including IFNγ, leading to the activation of antigen-presenting cells (APCs) and enhanced CD8+ effector T cell response [27], whereas Th2 CD4+ T cells can dampen the activity of APCs [28]. Regulatory T cells (Tregs), characterized by the CD4+FOXP3+ phenotype, counteract the effector T cell mechanisms via secretion of immunosuppressive cytokines like IL10 as well as by direct inhibition of APCs [29]. Tumor-associated macrophages (TAMs) and myeloid-derived suppressor cells (MDSCs) are key regulators of cancer-related inflammation and resistance to immunotherapy. The M1/M2 polarization of macrophages is differentiated by the TME, where the M2 phenotype is referred to as tumorigenic promoting tumor progression [30,31].

Immune checkpoints like CTLA-4, PD-1, and LAG-3 are responsible for the balance between co-stimulatory and inhibitory signals influencing T cell-mediated immune responses [32,33] and the maintenance of self-tolerance under normal physiological conditions. Chronic antigen exposure within TME can lead to T cell exhaustion, which is characterized by unresponsive T cells expressing multiple co-inhibitory receptors [34,35] e.g., PD-1 [36,37,38,39,40] on tumor-infiltrating lymphocytes (TILs). In addition, the enhanced PD-1 expression of TILs reflects the large proportion of intratumoral CD4+ regulatory T cells, as well as the anergic state of CD8+ T cells [34,36,41]. The finding that the blockade of PD-1, as well as CTLA-4, can restore the effector function of exhausted T cells, defined immune checkpoints as potential drug therapy targets.

### 2.2. Immune Checkpoint Inhibitors: The Mechanism of Action

CTLA-4 antibody ipilimumab was the first ICI therapy to be accepted for the treatment of cancer. Due to a more favorable toxicity profile and better response rates, anti-PD-(L)1 therapies have become the major ICI treatments. The mechanistic basis of responses to PD-1 or PD-L1 antibodies is in inhibiting interferon-induced adaptive immune resistance. Interferon production is increased when antigen presentation activates the T cell receptor (TCR), leading to activation of effector cells alongside the upregulation of PD-L1 and indoleamine-pyrrole 2,3-dioxygenase (IDO) on intratumoral cells [42]. In addition to the interferon-inducible expression of PD-L1, which is common in most cancer types, PD-L1 can be constitutively expressed through gene amplification and activation of oncogenic pathways related to the signal transducer and activator of transcription (STAT) proteins or other interferon-receptor downstream modulators, reflecting innate immune resistance [43,44,45,46]. 

**Figure 1:**
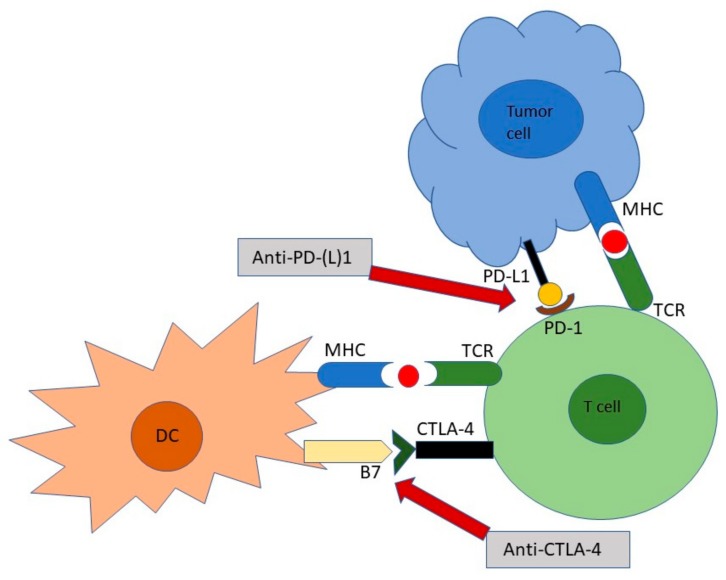
Mechanism of immune checkpoint blockade through CTLA-4 and PD-L1/PD-1 pathways.

After the PD-L1-PD-1-interaction (Figure 1), PD-1 can bind to SH2-domain-containing tyrosine phosphatase 1 (SHP-1) and 2, thus leading to dephosphorylation of key signaling intermediates, including kinases like Akt and PI3K, which might terminate early TCR signaling [47,48], and influence the duration of T cell-target cell contact [49]. The disruption of PD-L1-PD-1 interaction leads to diminished T cell movement, thus, providing the possibility for extended antigen-specific T cell and antigen-bearing APC interaction, which is critical for full T cell activation [50,51]. In contrast to CTLA4, which contributes to T cell activation centrally, PD-1 regulates effector T cell activity in peripheral tissues and tumors (Figure 1). Furthermore, PD-1 is more broadly expressed than CTLA-4 on other non-T lymphocyte subsets of immune cells [52,53]. 

## 3. Current Predictive Biomarkers for Immune Checkpoint Inhibitor Therapies

### 3.1. Tumor-Infiltrating Immune Cells

Cancer immunology is a very complex entity and, therefore, the characterized markers thus far have suboptimal predictive values. PD-L1 expression of tumor cells was the first predictive biomarker evaluated in clinical trials investigating the PD-(L)1 agents. It has been shown that the magnitude of pretreatment PD-L1 expression on tumor cells [12,54] and on immune cells [55] predicts clinical outcome on multiple tumor types. Though baseline PD-L1 expression is widely used as a biomarker in clinical practice, it is valid only in limited cancers such as NSCLC, urothelial, and head and neck (H&N) squamous cell cancers, and also, patients with PD-L1 negative tumors can benefit from PD-(L)1 inhibitor therapy. Furthermore, PD-L1 expression is dynamic and may change over the course of clinical treatment [56], raising questions over the rationale of the sole use of baseline expression. Not only the expression of PD-L1 but also the location and number of immune cells in the TME predicts treatment efficiency of ICIs. Pre-existing CD8+ TILs localized in the invasive tumor margin were predictive of melanoma response to PD-1 or CTLA-4 inhibitor therapy [57,58,59], whereas PD-L1+ melanocytes located next to TILs was shown to lead to the secretion of interferon-gamma as a form of adaptive immune resistance [60]. Two predictive classification schemas on the interactions between tumor and host immunity in the TME have been proposed. The first one is based on four different types of TME reflecting the PD-L1 status and the presence or absence of TILs [61], where the type I with TIL+/PD-L1+ is the most likely to respond to PD-(L)1 immune checkpoint blockade. The second framework classifies cancers into T cell-inflamed (also called “hot”) tumors versus non-inflamed (or “cold”) tumors [62], where the T cell-inflamed tumor-type contributes to a higher probability of response to ICIs.

### 3.2. Tumor Genetics

A clinically relevant, although rarely applicable biomarker used to predict outcome and guide patient selection for ICI therapy, is microsatellite instability (MSI), which is a condition of genetic hypermutability that results from impaired DNA mismatch repair (MMR). Approximately 5% of solid tumors are characterized by either the presence of MSI or by the absence of one or more MMR proteins, implying deficient (d)MMR status. MSI/dMMR status has been shown to poses positive predictive values for treatment benefit from the PD-1 blockade [63,64]. MSI is the first biomarker used to select patients for PD-1 inhibitor therapy irrelevant of tissue/tumor type with FDA approval of pembrolizumab after the first-line therapy for metastatic, MSI-high tumors.

Tumor mutation burden (TMB) and neoantigen load are biomarkers similar in concept to MSI, though, they have not been validated independently to predict response. TMB is a measurement of mutations carried by tumor cells [65]. There are data demonstrating the association of higher TMB to improved survival in patients receiving ICIs [66]. A recent study investigating TMB in NSCLC has shown that TMB can predict better outcomes of single-agent PD-1 treated patients but not for chemotherapy and PD-1 combination [67]. In clinical practice, the usability of TMB as a predictive biomarker has been scarce due to the somewhat variable definitions of high TMB, the price, and technical requirements of its’ determination. There is evolving data showing that both TMB and MSI status could be assessed from liquid biopsies, which probably will increase their diagnostic value in clinical practice [68]. 

Of the tumor single-gene alterations, the most prominent evidence of the predictive role exists in NSCLC with genes such as *STK11*, *KEAP1*, *K-Ras*, *EGFR*, and *ALK*. *STK11* is a tumor suppressor gene that is inactivated e.g., in NSCLC and melanoma [69,70], and in NSCLC, mutations in *STK11* have been shown to relate to a lack of response to immunotherapy. Studies have hypothesized that this might connect to a distinct immune environment linked to *STK11* tumors such as specific T cell excluded phenotypes [71,72,73]. Studies have shown the negative predictive value of *STK11* mutations in NSCLC for ICIs either in monotherapy or in combination with chemotherapy [74]. Based on recent data, *KEAP1* mutations among NSCLC patients are associated with inferior treatment responses to ICIs, despite other favorable molecular features such as high TMB [75]. *KRAS* mutation is the most common oncogenic aberration in NSCLC with up to 30% incidence in patients with adenocarcinoma in Western countries [76], and is associated with increased benefit from ICIs when it does not co-occur with *STK11* or *KEAP1* mutations. In general, the presence of *EGFR* mutations or *ALK* translocations in NSCLC is related to poor response to ICIs.

### 3.3. Circulating Markers

Systemic inflammation investigated using commonly characterized blood-based biomarkers has been shown to be related to the treatment response to ICIs. Elevated C-reactive protein (CRP) levels have been associated with poor responses to ICIs [77,78,79,80]. Other widely acknowledged prognostic markers for deleterious systemic inflammation include an elevated neutrophil-to-lymphocyte ratio (NLR) and lactate dehydrogenase (LDH). NLR is a marker for the general immune response to various stress stimuli, and it is shown to predict outcome among NSCLC and melanoma patients treated with PD-1 inhibitors [78,81,82,83,84], and CTLA-4 antibodies [78,83,85,86]. Raised LDH level is a classic inflammatory marker in patients with cancer. High baseline levels of LDH are linked to poor survival and inferior response to ICIs on melanoma and NSCLC patients [87,88].

Other potential soluble biomarkers include TCR diversity and clonality [89,90], as well as circulating immune cell subsets such as the number MDSCs or different T cell phenotypes [91,92,93]. The is evolving data on the negative prognostic meaning of PD-L1+ circulating tumor cells (CTCs) in NSCLC [94,95], however, the clinical benefit of immune checkpoint blockade in NSCLC based on PD-L1 status of circulating tumor cells remains uncertain. The prognostic role of soluble forms of PD-1 and PD-L1 (sPD-1, sPD-L1) on peripheral blood is unclear. There is data on the negative prognostic role of elevated serum sPD-L1 on stage IV melanoma [96], and NSCLC patients [97]. Still, findings from patients with pancreatic cancer suggest that sPD-1 and sPD-L1 are more indicators of systemic inflammation than a reflection of tumoral expression of PD-L1 [98], which could explain the dichotomy compared to the positive predictive role of high tissue PD-L1 expression.

### 3.4. The Prognostic Role of Gut Microbiota and Microbiome

The physiological importance of bacteria within the intestine, the microbiota, has been recognized through their effects on immune regulation, and pathogen niche exclusion [99,100]. There is evolving evidence that the gut microbiome has both prognostic and predictive value to treatment benefit from PD-(L)1 blockade [101,102,103,104], and in melanoma patients treated with ipilimumab [105]. According to the studies, significant differences were observed in the diversity and composition of the patient gut microbiome of responders versus non-responders. The imbalance in gut flora composition correlated with impaired immune cell activity in non-responders [104]. In addition, immune profiling suggested enhanced systemic and antitumor immunity in responding patients with a favorable gut microbiome [103]. The existing data creates a rationale for further studies in order to find ways to modulate the human microbiota therapeutically [106].

## 4. The Expanding Field of Cancer Immune Checkpoint Inhibitors

There is a constantly growing number of indications for ICIs in advanced cancers. Due to hundreds of studies already published of ICI monotherapies in an advanced disease setting, it is likely that the indications with the highest activity have already been discovered. ICI monotherapies are currently widely investigated in localized and locally advanced disease setting in adjuvant or neo-adjuvant schemes in multiple cancer types such as melanoma (NCT02977052; NCT04007588), NSCLC (NCT03425643; NCT02998528), and H&N SCC (NCT02296684; NCT03247712). Of the earlier disease settings, ICI monotherapies have been approved based on phase III disease-free survival (DFS) and/or overall survival (OS) evidence in the adjuvant treatment of high-risk melanoma, and as consolidation therapy after stage III NSCLC chemoirradiation [14,15,16]. Currently, published neo-adjuvant studies are generally small in sample size and pathological responses are often used as a primary endpoint [107,108,109]. It is unclear how good of a surrogate marker pathological response is for DFS or OS in the context of ICIs, even though a recent neoadjuvant trial on stage III melanoma patients showed a positive correlation between pathologic complete response and longer relapse-free survival after 18 months of follow-up [110]. However, it is likely that growing number of ICI therapies will be approved in adjuvant or neo-adjuvant setting in coming years.

## 5. Immune Checkpoint Inhibitor Combinations

**Figure 2:**
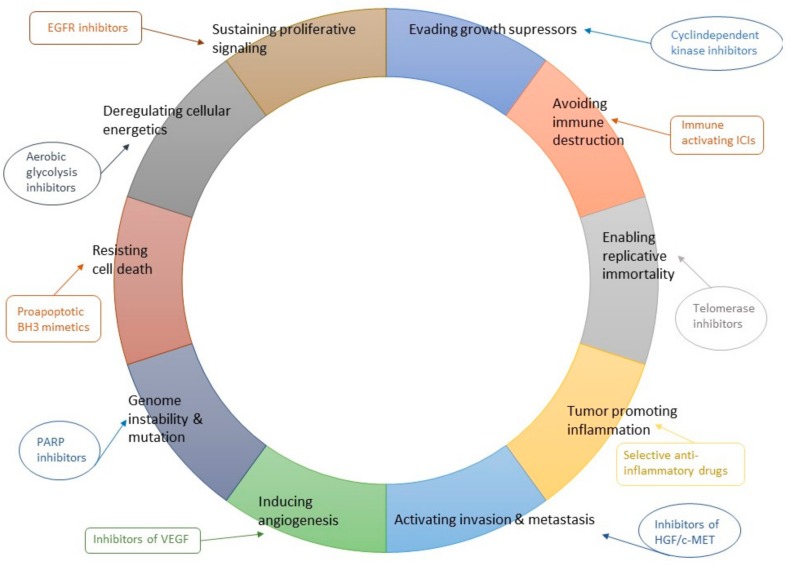
Therapeutic rationale of combinatory treatments based on hallmarks of cancer.

ICI monotherapy has limited activity in advanced cancers, leaving a wide room for therapeutic improvements. A current consensus on the landscape of immune-evasive tumors emphasizes the role of immunosuppressive T cell excluded TME with distinctive features of immune cell contextures and signaling pathway expressions [111]. Overall, therapeutic approaches aiming to overcome innate and adaptive ICI resistance most likely would increase ICIs therapeutic potential. However, tumor immunology is a very complex entity with individual differences both in host and tumor, and resistance mechanisms are poorly understood (Figure 2). Therefore, selecting the right combination of therapy for the right patient is very challenging. Currently, ICIs have mainly been investigated in combination with standard-of-care therapies in existing treatment schemes without strong proof of biological rationale and patient selection. ICIs are or have been investigated, at least, in combination with other ICIs (ICI-ICI), chemotherapy (ICI-chemotherapy), targeted cancer therapies (ICI-TKI), and radiotherapy [112]. At present, clinically the most relevant therapeutic uses for anti-PD-(L)1-anti-CTLA-4 antibody combinations are in advanced melanoma [113], RCC [114], and NSCLC [115], anti-PD-(L)1 antibody -chemo in lung cancers [116,117] and triple-negative breast cancer [118], and anti-PD-(L)1 antibody -TKI in RCC [119]. It is likely that we have just seen a glimpse of the therapeutic invasion of ICI combinations and numerous new indications will be approved in the future. There is a strong need for basic tumor immunology research, preclinical modeling of combinatory treatments with tumors closely resembling human cancers, and innovative clinical trials to fully unleash the potential of ICI combinations for the benefit of patients.

## 6. Special Populations

Phase III clinical trials with ICIs have generally excluded special populations from the studies, and data on their management is scarce. Few prospective trials with a restricted number of individuals have shown that ICIs can bring meaningful clinical benefits to patients not fulfilling the generic inclusion criteria. According to data based on small clinical trials, the safety of anti-PD-1 therapy seems to be similar in patients with performance status ECOG ≥2 but these patients do not derive the same clinical benefit as the ones with ECOG 0–1. However, the benefit or safety of PD-1s seems to be similar regardless of the patient’s age [120,121,122].

Another patient population typically excluded from clinical trials are patients with central nervous system (CNS) metastasis. Unique features of CNS such as the protecting blood–brain barrier, in addition to different tumor inflammatory microenvironments compared to extracranial disease sites [123,124], might prevent patients with brain metastasis from gaining the same clinical benefit from ICIs, and hence, most clinical trials have had brain metastasis as an exclusion criterion. Retrospectively, it has been shown that ICIs can have clinical activity with an intracranial response in brain metastases of melanoma and NSCLC [125,126]. Two prospective clinical trials with a limited number of subjects have suggested that ICI-ICI combination can bring meaningful clinical benefit (intracranial clinical benefit rate of ~50%) to asymptomatic melanoma patients with brain metastasis [127,128]. Based on existing data, it is hard to conclude whether and which patients with brains metastasis are candidates for ICI therapies in routine clinical practice.

Clinical data on cancer patients with autoimmune disease is inadequate as well. Underlining autoimmune disorders can worsen due to ICI initiation, and limited evidence exists on the treatment of these patients. Retrospective studies have shown that about half of the patients with autoimmune disorders experience a flare of their autoimmune disease after ICI initiation and that immunosuppressive therapy at baseline is associated with poorer outcomes [129,130]. The risks and benefits should be carefully weighed when initiating ICIs on patients with autoimmune disorders, and shared decision making is emphasized in order to meet the informed consent of a patient fully. 

## 7. Controlling Treatment-Related Side-Effects of Icis

Even though the toxicity profile of single-anti-PD-(L)1 therapy is rather benign with ~15% incidence of grade 3–4 immune-related adverse events (irAE) [2,5,6,8,9,10,20], the non-causal dependency on dosage and infusion frequency creates a unique spectrum of immune-related adverse events (irAEs). In addition, side-effects of ICIs can occur from virtually all of the organ systems and can occur very late, even months or years after treatment discontinuation. The treatment of side-effects consists of an assessment of symptom etiology, its´ NCI-CTCAE grade, and treatment of the side-effect by checkpoint inhibitor therapy discontinuation and initiation of immunosuppressive medications according to international guidelines [131,132]. Furthermore, anti-PD-(L)1-anti-CTLA-4 antibody combination therapies have a much more toxic nature of around 30–50% of patients experiencing severe side-effects [133,134], whereas with anti-PD-(L)1-chemo combination (e.g., atezolizumab and nab-paclitaxel; pembrolizumab-cisplatin/carboplatin-pemetrexed; pembrolizumab-carboplatin plus paclitaxel or nab-paclitaxel) the rate of irAEs follows the toxicity profile of anti-PD-(L)1 monotherapies excluding renal treatment-related side-effects [116,117,118]. For the optimal follow-up of patients receiving a checkpoint inhibitor, there is a need for comprehensive assessment, grading, and long-term surveillance of symptoms.

Patient-reported outcomes (PROs) consist of health-related questionnaires filled by the patients themselves, which can capture symptoms and signs and their severity. Scheduled electronic (e) PROs have many advantages compared to paper questionnaires such as reducing timely and locational limitations and offering a continuous collection of symptoms in a cost-effective manner [135,136,137]. The addition of ePRO to standard follow-up has been shown to improve survival and QoL of cancer patients treated with chemotherapy [138,139], but at present, there is only limited retrospective data on ePRO follow-up of cancer patients receiving ICIs [140], even though some prospective trials are ongoing. Taken into account the somewhat unpredictable nature of irAEs, the possibility of coupling ePROs to urgency algorithm, which sends an alert to the care unit on severe or altering symptoms enabling rapid reaction and treatment of important medical events, makes ePRO follow-up of patients receiving ICIs even more plausible.

## 8. Economic Sustainability

ICI therapies present a substantial economic challenge, especially considering the lack of good predictive biomarkers and undefined optimal treatment duration. Furthermore, the growing number of indications and ICIs investigated in combinations leading to longer treatment periods compared to monotherapy, will result in a high economic burden. Cost-effectiveness analysis has been carried out in numerous indications for ICIs yielding cost-effectiveness in some indications with a high willingness-to-pay (WTP) threshold [141]. With a WTP threshold of $100,000/QALY, the first line monotherapy studies have shown the cost-effectiveness of the therapy in melanoma (nivolumab/pembrolizumab vs. dacarbazine/ipilimumab [142,143,144,145]) and in NSCLC PD-L1 ≥50% (pembrolizumab vs. chemotherapy [146]). Conversely, none of the combinations tested in NSCLC (pembrolizumab+chemotherapy vs. chemotherapy [147] or atezolizumab+bevazicumab+chemotherapy vs. bevazicumab+chemotherapy [148]) or melanoma (nivolumab-ipilimumab vs. nivolumab [149]) were cost-effective with ICER of $147,366–454,092/QALY.

The cost-effectiveness of ICIs could be improved by better patient selection, determination of the optimal treatment length, or decreasing direct drug costs by lowering the prices or by risk-sharing price models. Thus far, clinical trials have rather suboptimally defined the patient population most likely to benefit from ICI therapy. Furthermore, most of the clinical trials have not questioned shorter than disease progression approaches. Currently, only one clinical trial has investigated discontinuing anti-PD-1 therapy at a 1-year fixed time compared to continuous treatment. The results of the study showed that PFS was inferior for patients whose therapy was discontinued but OS was similar [150]. In addition, data from retrospective studies have shown that discontinuing anti-PD-1 therapy in response does not lead to rapid disease progression [151,152,153]. However, there is a strong need for high-quality evidence to define early stopping rules. Hence, two prospective trials, STOP-GAP (NCT02821013) and DANTE (ISRCTN15837212), are recruiting metastatic melanoma patients to evaluate the optimal treatment duration and the role of re-challenge of anti-PD-1 therapy in order to define whether we are over-treating with checkpoint inhibitors.

## 9. Discussion

In a short period of time, ICI therapies have changed how we treat numerous cancers, but we are, however, far from optimal ICI therapeutic use. In most indications, the majority of patients fail to respond, good predictive markers are lacking, and ICI therapies generate a marked economic burden. These hurdles demand new solutions to improve the clinical efficiency of ICIs. The clinical efficiency of ICIs could be improved by means such as better patient selection and side effect management, earlier disease settings, combinatory therapeutic approaches, and addressing treatment length for better economic sustainability.
